# Sarcopenia Predicts Mortality and Hepatic Encephalopathy After TIPS in Older Adults With Cirrhosis and Improves Prognostic Scores

**DOI:** 10.1111/liv.70733

**Published:** 2026-06-07

**Authors:** Dario Saltini, Silvia Nardelli, Roberto Miraglia, Davide Roccarina, Stefania Gioia, Federico Banchelli, Luigi Maruzzelli, Cristian Caporali, Gianmarco Falcone, Marcello Bianchini, Tomas Guasconi, Angelica Ingravallo, Simone Di Cola, Rosina Maria Critelli, Fabiola Milosa, Antonio Piscopo, Federico Casari, Andrea Salomé Velasco Mayorga, Filippo Scianò, Giovanni Battinelli, Francesco Ascari, Oliviero Riggio, Fabio Marra, Manuela Merli, Francesco Vizzutti, Filippo Schepis

**Affiliations:** ^1^ Severe Liver Diseases (M.E.C.) Departmental Unit, Department of Medical Specialties University Hospital of Modena “Policlinico”, University of Modena and Reggio Emilia, European Reference Network on Rare Liver Disorders (ERN Rare‐Liver) Modena Italy; ^2^ Department of Translational and Precision Medicine Sapienza University of Rome Rome Italy; ^3^ IRCCS ISMETT, Radiology Service Palermo Italy; ^4^ UPMC Italy Palermo Italy; ^5^ Department of Experimental and Clinical Medicine University of Florence Florence Italy; ^6^ Medical Statistics Unit, Department of Medical and Surgical Sciences University of Modena and Reggio Emilia Modena Italy; ^7^ Division of Interventional Radiology University Hospital of Modena “Policlinico” Modena Italy; ^8^ Department of Radiology, Interventional Radiology Unit Careggi Hospital Florence Italy; ^9^ EpatoGastro Lab – RBA‐Labs CHIMOMO Department University of Modena and Reggio Emilia Modena Italy; ^10^ Gastroenterology Unit University Hospital of Modena “Policlinico”, University of Modena and Reggio Emilia Modena Italy; ^11^ Portal Hypertension Departmental Unit, Department of Experimental and Clinical Medicine University of Florence Florence Italy

**Keywords:** aged, hepatic encephalopathy, liver cirrhosis, portosystemic shunt, prognostic models, skeletal muscle index

## Abstract

**Background and Aims:**

The role of transjugular intrahepatic portosystemic shunt (TIPS) in older adults remains controversial because of limited risk‐stratification tools. We aimed to assess whether sarcopenia and myosteatosis are independently associated with post‐TIPS mortality and overt hepatic encephalopathy (OHE) in patients aged ≥ 70 years, and whether adding sarcopenia to established prognostic scores improves discrimination for post‐TIPS mortality.

**Methods:**

This multicenter retrospective study included 115 consecutive patients with cirrhosis aged ≥ 70 years undergoing TIPS for refractory ascites or secondary prophylaxis of variceal bleeding. Sarcopenia and myosteatosis were assessed by computed tomography at L3. Post‐TIPS mortality and time to first OHE episode were analysed using Kaplan–Meier and Cox regression. Sarcopenia was integrated into established prognostic scores, and predictive performance was evaluated using time‐dependent ROC analyses.

**Results:**

Sarcopenia and myosteatosis were present in 60% and 80% of patients, respectively. During follow‐up, 49% died and 45% developed OHE. Sarcopenia was independently associated with both mortality and OHE, whereas myosteatosis and adipose‐tissue indices were not. Incorporating sarcopenia improved the discriminative performance of all scores, with MELD 3.0–sarcopenia showing the highest accuracy (AUC 0.845). Predicted survival probabilities clearly separated patients across MELD 3.0 categories according to sarcopenia status. For OHE, sarcopenia increased the risk while underdilated TIPS was protective, defining four distinct risk profiles.

**Conclusions:**

Sarcopenia is highly prevalent and independently predicts both mortality and OHE after TIPS in older adults. Its integration into prognostic tools enhances risk stratification and supports individualised decision‐making in this vulnerable population.

## Highlights

This study provides a scientific rationale for incorporating sarcopenia assessment into pre‐TIPS evaluation in patients aged ≥ 70 years, showing that reduced muscle mass independently predicts mortality and overt hepatic encephalopathy and improves the prognostic performance of standard liver‐severity scores. These findings are relevant for clinicians managing older adults with cirrhosis, in whom risk stratification before TIPS remains challenging. Integrating sarcopenia assessment into routine evaluation and procedural planning may support individualised decision‐making and patient counselling, although prospective validation is needed to confirm these results.

## Lay Summary

In older patients with cirrhosis being considered for a TIPS procedure, low muscle mass (sarcopenia) measured on a routine pre‐procedural CT scan independently predicts the risk of dying and of developing hepatic encephalopathy after TIPS. Adding this simple measurement to existing risk scores improves their accuracy and may help clinicians and patients make more informed decisions.

**Abbreviations**
AUROC: area under the receiver operating characteristic curve
CP: Child Pugh
CT: computed tomography
ExPeCT: Elderly Patients Calculator TIPS
FIPS: Freiburg index of post‐TIPS survival
HCC: hepatocellular carcinoma
HR: hazard ratio
INR: international normalised ratio
IQR: interquartile range
LT: liver transplantation
MALSD: metabolic dysfunction‐associated steatotic liver disease
MELD: Model for End‐Stage Liver Disease
OHE: overt hepatic encephalopathy
PCPG: portocaval pressure gradient
RA: refractory ascites
RI‐TIPS: Italian TIPS registry
ROC: receiver operating characteristic
SATI: subcutaneous adipose tissue index
SD: standard deviation
TIPS: transjugular intrahepatic portosystemic shunt
VATI: visceral adipose tissue index

## 1 Introduction

The concept of age‐based absolute contraindications has increasingly been questioned. Recent studies indicate that older adults may benefit from transjugular intrahepatic portosystemic shunt (TIPS), a well‐established treatment for complications of portal hypertension (PH) [1, 2, 3]. However, favourable outcomes depend on meticulous patient selection. Although biochemical parameters, such as creatinine and serum sodium levels, have been used to guide candidate selection, accurate risk stratification remains limited [4]. The survival benefit of TIPS in older adults has not been thoroughly characterised, and the risk of shunt‐related complications often complicates decision‐making, especially given the frequent unavailability of a transplant lifeline for this population [5].

Body composition has gained increasing attention as a prognostic factor in cirrhosis [6, 7, 8]. Sarcopenia [9]—characterised by reduced muscle mass, strength, and function—and myosteatosis [10]—a pathological fat infiltration into skeletal muscle—are highly prevalent in patients with cirrhosis [11] and contribute to morbidity and mortality [12, 13]. Moreover, both are intrinsically linked to aging [14, 15], further complicating their interpretation in older patients with cirrhosis. In the TIPS setting, sarcopenia may be particularly relevant: skeletal muscle is the primary extrahepatic site of ammonia metabolism, and reduced muscle reserve may impair the ability to tolerate the abrupt hemodynamic and metabolic changes induced by TIPS—a vulnerability magnified in older adults. Despite these considerations, data on body composition in TIPS candidates [16, 17, 18, 19, 20], particularly in older adults [21], remain limited.

The primary objective of this study was to assess whether sarcopenia and myosteatosis are independently associated with post‐TIPS mortality and time to first overt hepatic encephalopathy (OHE) episode in older adults with cirrhosis. The secondary objective was to evaluate whether incorporating sarcopenia into established prognostic scores improves discrimination for post‐TIPS mortality. As pre‐specified exploratory analyses, we examined predicted survival and OHE‐free survival profiles defined by sarcopenia status combined with key clinical covariates.

## 2 Patients and Methods

We conducted a multicenter retrospective study on consecutive patients aged 70 years or older from the Italian TIPS registry (RI‐TIPS) who underwent TIPS for refractory ascites (RA) or secondary prophylaxis of PH‐related variceal bleeding at one of the four participating centers (Florence, Modena, Palermo, and Rome) between June 2015 and March 2023. Cirrhosis diagnosis was based on clinical history, histology, or morphological imaging features, and an abdominal computed tomography (CT) scan within 3 months before TIPS was required. Beyond clinical contraindications to TIPS [22], exclusion criteria were: (a) hepatocellular carcinoma (HCC) or any other active malignant neoplasm, with the exception of non‐melanoma skin cancers (basal‐ or squamous‐cell carcinomas, given their high prevalence in older adults, negligible impact on survival and body composition, and inconsistent reporting in RI‐TIPS); (b) active alcohol use per addiction‐center criteria; (c) concomitant neuromuscular disease; (d) loss to 6‐month follow‐up, unless liver transplant (LT) or death occurred; and (e) CT scan considered inadequate for muscle assessment.

TIPS was performed as previously described [23]. During the study period, patients were treated with first‐generation VIATORR endoprosthesis until 2018, followed by the new‐generation VCX (W.L. Gore, Flagstaff, AZ). All TIPS procedures were performed under deep sedation using remifentanil and midazolam. Porto‐caval pressure gradient (PCPG) was measured before and after TIPS, in accordance with both Italian [22] and international guidelines [24]. Before TIPS, the patients were treated with non‐selective beta‐blockers according to international guidelines [24]. Clinical and laboratory data—including age, sex, aetiology of liver disease, indication for TIPS, history of OHE and clinical ascites, comorbidities, biochemistry within 5 days before TIPS (serum bilirubin, albumin, creatinine, sodium, international normalised ratio [INR], white blood cells and platelet count), stent‐graft generation/diameter, and PCPG values—were recorded in an anonymised dedicated case report form. Prognostic scores—Model for End‐Stage Liver Disease‐Sodium (MELD) [25], MELD‐Na, MELD 3.0 [26], Child–Pugh score (CP), Freiburg index of post‐TIPS survival (FIPS) [27], and Elderly Patients Calculator TIPS (ExPecT) [1], were calculated according to previously reported formulas (Supporting Information).

Based on the RI‐TIPS protocol, no patients received primary OHE prophylaxis before or after TIPS; patients with prior OHE received secondary prophylaxis with oral non‐absorbable disaccharides ± rifaximin. Patients were followed up according to the RI‐TIPS protocol, with visits to the outpatient clinic scheduled at 1, 3, and 6‐months post‐TIPS, then every 6 months or as clinically indicated, until death or LT. At each visit, the same medical team assessed all patients for OHE, defined as grade II or higher on the West‐Haven scale; only patients developing post‐TIPS OHE started pharmacological prophylaxis according to international guidance [24]. Patients and caregivers were thoroughly educated on early OHE recognition and instructed to promptly notify the medical team for confirmation and hospitalisation.

The RI‐TIPS survey was approved by the Ethical Committee of the University of Florence (lead‐center protocol 93/2012) and by the ethical committees of all participating centers. This study complied with the Declaration of Helsinki; all patients provided data at the time of TIPS placement.

### 2.1 Muscle Changes Evaluation

CT images were transmitted to the referral center for muscle changes analysis (Rome), where two trained experts (98% interobserver correlation) used SliceOmatic V4.2 (Tomovision) to assess sarcopenia and myosteatosis. Total cross‐sectional area of the abdominal skeletal muscle and fat mass were evaluated at the L3 vertebra (psoas, paraspinal, and abdominal‐wall muscles, including rectus abdominis, transverse abdominis, and internal/external oblique). Muscle area was normalised for stature, resulting in L3 skeletal mass index (SMI, cm^2^/m^2^). Sarcopenia was defined as SMI < 50 cm^2^/m^2^ in men and < 39 cm^2^/m^2^ in women [28]. Myosteatosis was defined by mean muscle attenuation (MA) at the same image, using mortality‐associated cut‐offs: < 41 HU in patients with a body mass index (BMI) < 25 kg/m^2^ and < 33 HU for BMI ≥ 25 kg/m^2^ [29]. Subcutaneous (SAT) and visceral (VAT) adipose tissue were defined by HU thresholds (−190 to −30 and −150 to −50, respectively) and normalised to stature, yielding the subcutaneous (SATI) and visceral (VATI) adipose tissue indices (cm^2^/m^2^) [30].

### 2.2 Statistical Analysis

The two co‐primary outcomes were post‐TIPS all‐cause mortality (over total follow‐up) and time to first OHE episode after TIPS. The secondary outcome was the discriminative performance of sarcopenia‐augmented prognostic scores for post‐TIPS mortality. Analyses of predicted survival and OHE‐free survival profiles by clinically relevant covariate combinations were pre‐specified as exploratory and intended for descriptive risk stratification rather than for formal model derivation.

Continuous variables are reported as mean ± SD or median (IQR), and compared using Student's *t*‐test or the Mann–Whitney *U* test; categorical variables were compared with the chi‐squared or Fisher's exact test. Correlations between non‐normally distributed continuous variables were assessed using Spearman's rank coefficient. Time‐to‐event outcomes were analysed by Kaplan–Meier and log‐rank test, with follow‐up calculated from TIPS to last contact, death or LT. No specific time horizon was prespecified; 12‐ and 24‐month probabilities are reported as descriptive estimates. For OHE, time to first episode was considered, censoring at death or last follow‐up. Risk factors for both outcomes were evaluated using Cox proportional hazards models; variables with *p* < 0.10 in univariable analysis, plus clinically relevant covariates (sex and history of ascites), entered multivariable models. To address death as a competing event for OHE, cumulative incidence functions (Aalen–Johansen) and Fine–Grey subdistribution hazards models were applied. Because OHE prophylaxis was administered only to patients with prior OHE (RI‐TIPS protocol), prior OHE and HE prophylaxis were considered collinear and not entered simultaneously. Sarcopenia was integrated into prognostic scores by two approaches: for MELD‐based scores (MELD, MELD‐Na, and MELD 3.0), sarcopenia was integrated using a previously validated formula [7], with sarcopenia coded as a binary variable (0 = absent, 1 = present); for non–MELD‐based scores (CP, FIPS, and ExPecT), sarcopenia was incorporated as a binary additive component (score + sarcopenia). This pragmatic strategy avoided recalibration or re‐estimation of the score coefficients and maintained comparability with the original validated models. Discriminative performance was evaluated using time‐dependent receiver operating characteristic (ROC) curves, calculated with the *timeROC* package, areas under the curve (AUCs) compared with DeLong's test, and Harrell's concordance index (C‐index). Predicted survival probabilities for clinically relevant risk profiles were derived from Cox models. Analyses were performed using R software (R Foundation for Statistical Computing, Vienna, Austria).

## 3 Results

### 3.1 Characteristics of Patients

Of 516 patients with cirrhosis undergoing TIPS at the participating centers, 182 were aged ≥ 70 years; 134 met the indications for refractory ascites or secondary prophylaxis. After exclusion of patients with inadequate baseline CT (*n* = 7) or loss to follow‐up (*n* = 12), 115 patients were included (Figure S1). Baseline characteristics are shown in Table 1. Median age was 74 years (IQR 72–77); 62% were male. TIPS was performed for RA in 57% and for secondary prophylaxis of PH‐related bleeding in 43% of cases; 52% received under‐dilated TIPS (dilation diameter ≤ 7 mm). Median CP score was 8 (7, 8) and median MELD, MELD‐Na, and MELD 3.0 scores were 11 (10–15), 13 (11–17), and 14 (11–18), respectively. Sarcopenia was observed in 60% (mean SMI 42.5 ± 7.4 cm^2^/m^2^ for females, 44.0 ± 7.7 cm^2^/m^2^ for males) and myosteatosis in 80% (mean MA 31.8 ± 8.0 HU for BMI < 25 kg/m^2^ and 27.4 ± 7.8 HU for BMI ≥ 25 kg/m^2^). A combined analysis of sarcopenia and myosteatosis showed that only 8% had neither condition, 32% had myosteatosis without sarcopenia, 12% sarcopenia without myosteatosis and 48% both (Figure S2). Sarcopenic and non‐sarcopenic patients did not differ in liver disease aetiology, TIPS indications, liver function, or hemodynamic parameters (Table 1). Sarcopenia was significantly more prevalent in male patients (70% vs. 50%, *p* = 0.034), and sarcopenic patients had a lower baseline median dry BMI (23.8 vs. 27.2 kg/m^2^, *p* < 0.001). Myosteatosis prevalence was similar across sarcopenia status (*p* = 0.924). Sarcopenic patients had reduced VATI [22.8 (IQR 10.6–43.6) vs. 42.2 (IQR 21.8–58.7) cm^2^/m^2^; *p* < 0.001] and SATI [32.9 (IQR 20.7–58.3) vs. 69.3 (IQR 43.3–85.1) cm^2^/m^2^; *p* < 0.001]. Baseline SMI was similar between RA and PH‐related bleeding indications (42.1 ± 7.6 vs. 42.9 ± 8.1 cm^2^/m^2^; *p* = 0.488), while MA was slightly lower in the RA group (28.6 ± 8.4 vs. 31.3 ± 7.6 HU; *p* = 0.053). Both SATI [38.7 (IQR 21.9–61.6) vs. 64.4 (IQR 36.3–88.0) cm^2^/m^2^; *p* = 0.002] and VATI [23.5 (IQR 13.5–40.9) vs. 44.9 (IQR 17.8–57.7) cm^2^/m^2^; *p* = 0.016] were significantly lower in patients with RA. Adipose tissue indices showed weak‐to‐moderate positive correlations with SMI, whereas SATI, but not VATI, was inversely correlated with MA (Figure S3).

**TABLE 1:** Clinical, biochemical, muscle and adipose tissue parameters for the entire cohort and stratified according to the presence of sarcopenia.

	No sarcopenia (*N* = 46)	Sarcopenia (*N* = 69)	Total (*N* = 115)	*p*
Male sex, *n* (%)	23 (50%)	48 (70%)	71 (62%)	0.034
Age, years (IQR)	73 (71, 76)	74 (72, 77)	74 (72, 77)	0.070
Dry BMI, kg^2^/m^2^, (IQR)	27.2 (24.2, 29.4)	23.8 (22.4, 25.6)	24.6 (22.6, 27.8)	< 0.001
Patients with obesity, *n* (%)	10 (22%)	4 (6%)	14 (12%)	0.010
SMI, cm^2^/m^2^, (±SD)	48.1 (6.6)	38.7 (6.1)	42.5 (7.8)	< 0.001
Muscle Attenuation, HU (±SD)	27.5 (8.8)	31.3 (7.4)	29.8 (8.1)	0.026
Myosteatosis, *n* (%)	37 (80.4%)	55 (79.7%)	92 (80.0%)	0.924
SATI, cm^2^/m^2^, (IQR)	69.3 (43.3, 85.1)	32.9 (20.7, 58.3)	45.9 (27.0, 75.2)	< 0.001
VATI, cm^2^/m^2^, (IQR)	42.2 (21.8, 58.7)	22.8 (10.6, 43.6)	30.4 (15.7, 49.9)	< 0.001
Liver disease aetiology				0.941
ALD, *n* (%)	9 (20%)	15 (22%)	24 (21%)	
Viral‐related, *n* (%)	20 (43%)	28 (40%)	48 (42%)	
MASLD, *n* (%)	17 (37%)	26 (38%)	43 (37%)	
Diabetes, *n* (%)	20 (44%)	25 (36%)	45 (39%)	0.435
Chronic kidney disease, *n* (%)	9 (20%)	7 (10%)	16 (14%)	0.153
Prior OHE, *n* (%)	9 (20%)	10 (15%)	19 (17%)	0.473
Lactulose prophylaxis, *n* (%)	9 (20%)	9 (13%)	18 (%)	0.434
Rifaximin prophylaxis, *n* (%)	7 (15%)	10 (15%)	17 (%)	1.000
History of ascites^a^, *n* (%)	25 (54%)	49 (71%)	74 (64%)	0.068
Oesophageal varices, *n* (%)	37 (80%)	56 (81%)	93 (81%)	0.923
TIPS main indication				0.443
Refractory ascites, *n* (%)	24 (52%)	41 (59%)	65 (57%)	
II prophylaxis of VB, *n* (%)	22 (48%)	28 (41%)	50 (43%)	
TIPS underdilated^b^, *n* (%)	28 (61%)	32 (46%)	60 (52%)	0.127
Post‐TIPS PCPG < 12 mmHg, *n* (%)	31 (67%)	41 (59%)	72 (63%)	0.387
Post‐TIPS PCPG < 10 mmHg, *n* (%)	27 (59%)	31 (45%)	58 (50%)	0.148
PLTs count, 10^9^/L (IQR)	92 (65, 124)	81 (58, 122)	87 (58, 123)	0.477
Albumin, g/L (±SD)	3.1 (0.6)	3.2 (0.6)	3.2 (0.6)	0.247
Total bilirubin, mg/dL (IQR)	1.25 (0.88, 1.78)	1.21 (0.88, 1.67)	1.21 (0.88, 1.71)	0.882
INR (IQR)	1.25 (1.19, 1.39)	1.23 (1.10, 1.39)	1.23 (1.14, 1.40)	0.220
Creatinine, mg/dL (IQR)	1.11 (0.80, 1.37)	1.15 (0.93, 1.50)	1.15 (0.84, 1.42)	0.282
Serum sodium, mmol/L (±SD)	138 (4)	137 (5)	137 (5)	0.319
CP score (IQR)	8 (7, 9)	8 (7, 8)	8 (7, 8)	0.660
CP class				0.559
A	9 (20%)	16 (23%)	25 (22%)	
B	35 (76%)	47 (68%)	82 (71%)	
C	2 (4%)	6 (9%)	8 (7%)	
MELD score (IQR)	11.5 (9.9, 14.5)	11.4 (10.0, 14.6)	11.4 (9.9, 14.5)	0.984
MELD‐Na score (IQR)	13.0 (10.4, 15.1)	13.0 (10.6, 17.8)	13.0 (10.5, 17.0)	0.644
MELD 3.0 score (IQR)	13.8 (11.3, 15.4)	13.5 (10.7, 18.3)	13.5 (10.8, 17.6)	0.833
ExPeCT score (IQR)	−0.51 (−0.79, −0.30)	−0.48 (−0.71, −0.18)	−0.48 (−0.76, −0.26)	0.215
FIPS score (IQR)	0.42 (0.23, 0.59)	0.42 (0.11, 0.79)	0.42 (0.12, 0.71)	0.786

Abbreviations: BMI, body mass index; CP, Child Pugh; ExPeCT, Elderly Patients Calculator TIPS; FIPS, Freiburg index of post‐TIPS survival; HU, Hounsfield unit; INR, international normalised ratio; IQR, interquartile range; IQR, interquartile range; MASLD, metabolic dysfunction‐associated steatotic liver disease; MELD, Model for End‐Stage Liver Disease; OHE, overt hepatic encephalopathy; PCPG, portocaval pressure gradient; SATI, subcutaneous adipose tissue index; SD, standard deviation; SMI, skeletal muscle index; TIPS, transjugular intrahepatic portosystemic shunt; VATI, visceral adipose tissue index.

^a^ Ascites grade ≥ 2.

^b^ TIPS were defined “underdilated” when dilated with an angioplasty balloon‐catheter with a diameter less than or equal to 7 mm.

### 3.2 Predictors of Post‐TIPS Mortality

**FIGURE 1:**
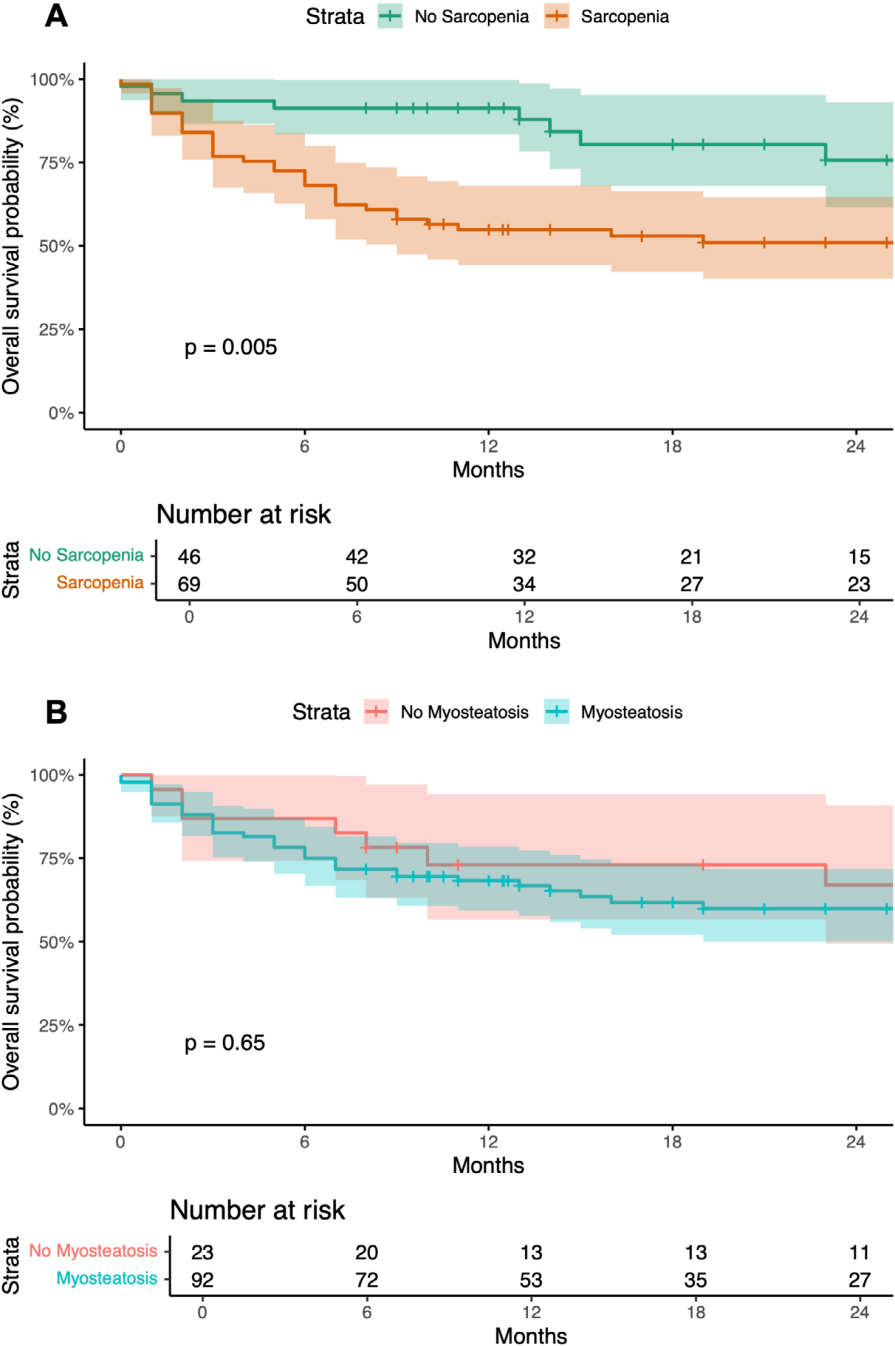
Kaplan–Meier estimates of post‐TIPS overall survival stratified by the presence of sarcopenia (A) and myosteatosis (B). Differences between the groups were assessed using the log‐rank test. TIPS, transjugular intrahepatic portosystemic shunt.

During a median follow‐up of 13 months (IQR 7–29), 56 patients (49%) died and none underwent LT. Median post‐TIPS survival was 36 months (IQR 7–68); 12‐ and 24‐month survival probabilities were 69% (95% CI 61–78) and 61% (95% CI 52–72), respectively (Figure S4). Sarcopenic patients had reduced 12‐month post‐TIPS survival (55% vs. 91%; log‐rank *p* = 0.005), whereas myosteatosis was not associated with survival (73% vs. 68%; log‐rank *p* = 0.650) (Figure 1). In univariate Cox analysis (Table S1), liver dysfunction (total bilirubin and INR), renal‐function indicators (creatinine, serum sodium), and prognostic scores (MELD, MELD‐Na, MELD 3.0, CP, FIPS, ExPeCT) were significantly associated with post‐TIPS mortality; sarcopenia, but not myosteatosis, was among the strongest predictors. Sarcopenia remained independently associated with mortality in multivariable models including selected clinically relevant covariates (sex, history of clinical ascites) and after addition to MELD 3.0, FIPS and CP scores, confirming its independent prognostic role (Table 2).

**TABLE 2:** Multivariable Cox regression models assessing independent predictors of post‐TIPS mortality.

Variable	Multivariable analysis—individual variables		Multivariable analysis—MELD 3.0		Multivariable analysis—CP score		Multivariable analysis—FIPS	
	HR (95% CI)	*p*	HR (95% CI)	*p*	HR (95% CI)	*p*	HR (95% CI)	*p*
Male sex	1.08 (0.59–1.96)	0.810	—	—	0.99 (0.56–1.77)	0.978	1.05 (0.58–1.87)	0.880
Sarcopenia	**2.39 (1.26–4.54)**	**0.008**	**2.14 (1.15–3.96)**	**0.016**	**2.21 (1.17–4.21)**	**0.015**	**2.14 (1.14–4.01)**	**0.018**
History of ascites^a^	1.63 (0.82–3.24)	0.160	1.32 (0.69–2.54)	0.402	**—**	**—**	**—**	**—**
Total Bilirubin, mg/dL	1.31 (0.96–1.80)	0.088	**—**	**—**	**—**	**—**	**—**	**—**
INR	**1.21 (1.07–1.36)**	**0.003**	**—**	**—**	**—**	**—**	**—**	**—**
Creatinine, mg/dL	1.19 (0.66–2.17)	0.563	**—**	**—**	1.22 (0.67–2.23)	0.516	—	—
Sodium, mmol/mol	**—**	**—**	**—**	**—**	**—**	**—**	0.99 (0.92–1.05)	0.687
CP score	**—**	**—**	**—**	**—**	**1.32 (1.06–1.63)**	**0.013**	**—**	**—**
MELD 3.0 score	**—**	**—**	**1.09 (1.04–1.14)**	**< 0.001**	**—**	**—**	**—**	**—**
FIPS score	**—**	**—**	**—**	**—**	**—**	**—**	**2.43 (1.33–4.46)**	**0.004**

*Note:* Cox proportional hazards regression was used to explore associations between baseline variables and the risk of post‐TIPS mortality. For each variable, hazard ratios (HR), 95% confidence intervals (CI), and *p* values are reported. Values in bold indicate statistically significant independent predictors (*p* < 0.05). Prognostic scores were entered individually to avoid collinearity.

Abbreviations: CI, confidence interval; CP, Child Pugh; FIPS, Freiburg index of post‐TIPS survival; HR, hazard ratio; INR, international normalised ratio; MELD, Model for End‐Stage Liver Disease; TIPS, transjugular intrahepatic portosystemic shunt.

^a^ Ascites grade ≥ 2.

### 3.3 Predictive Performance of Prognostic Scores and Impact of Sarcopenia

**FIGURE 2:**
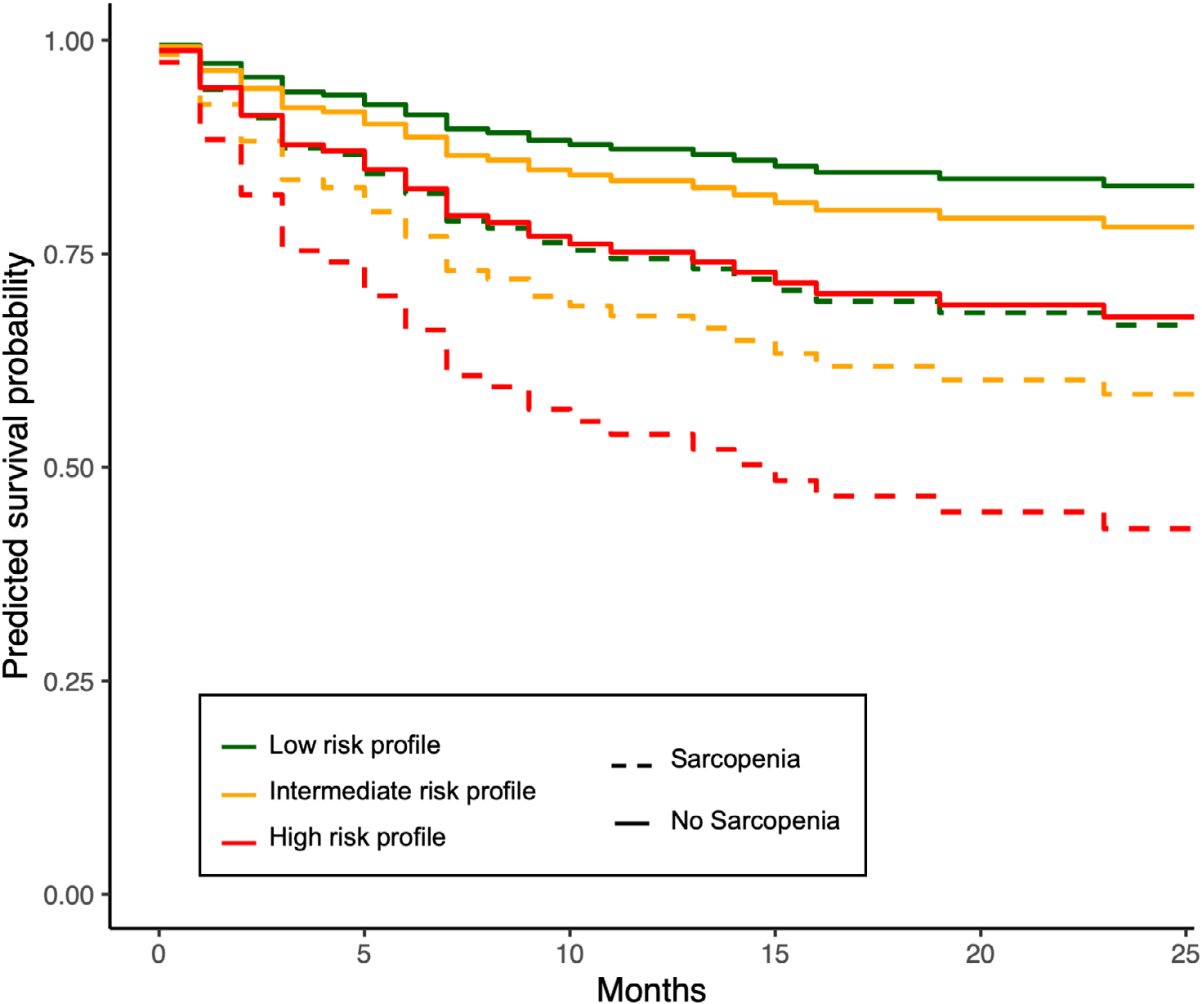
Predicted post‐TIPS survival probabilities in older adult patients according to three MELD 3.0–based risk profiles. Green curve: Low risk (MELD 3.0 = 10); yellow curve: Intermediate risk (MELD 3.0 = 13); red curve: High risk (MELD 3.0 = 18). Dashed lines indicate the presence of sarcopenia, while solid lines indicate the absence of sarcopenia. MELD, Model for End‐Stage Liver Disease; TIPS, transjugular intrahepatic portosystemic shunt.

**FIGURE 3:**
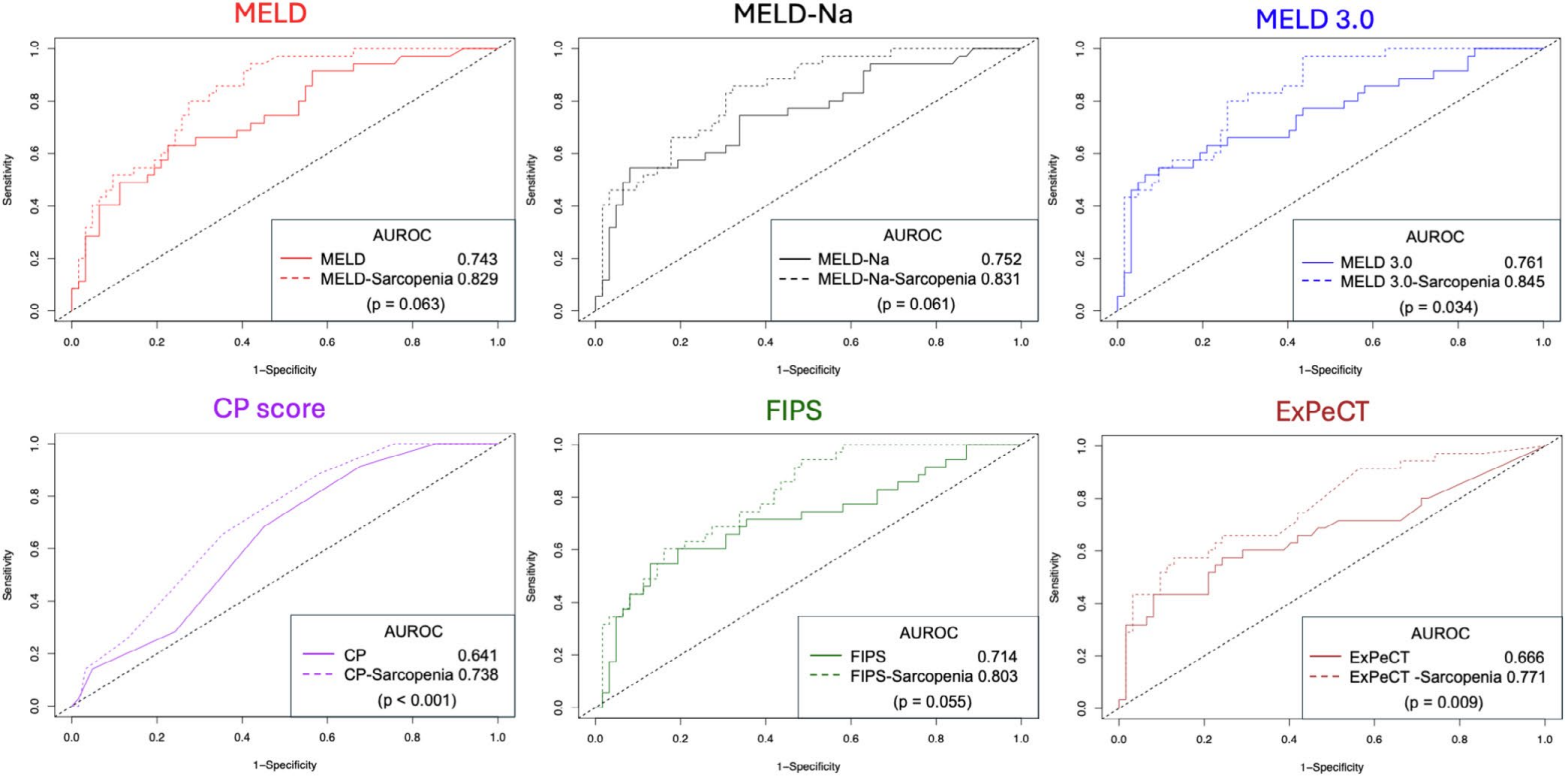
Time‐dependent ROC curves for 1‐year post‐TIPS mortality in older adult patients, comparing established prognostic scores with their sarcopenia‐augmented counterparts (dashed lines). Differences between AUROC were assessed using DeLong's test. AUROC, area under the receiver operating characteristic curve; CP, Child–Pugh score; ExPeCT, Elderly Patients Calculator TIPS; FIPS, Freiburg index of post‐TIPS survival; MELD, Model for End‐Stage Liver Disease; TIPS, transjugular intrahepatic portosystemic shunt.

In an exploratory analysis, predicted survival probabilities according to three MELD 3.0‐based risk profiles (low: 10, intermediate: 13, and high: 18) showed that within each group, sarcopenia was consistently associated with lower predicted survival at 12 and 24 months (Figure 2; Table S2), refining risk stratification beyond liver‐disease severity alone. Established scores showed moderate‐to‐good discrimination at 12 months (AUC 0.641‐0.761; Figure 3). Addition of sarcopenia significantly improved discrimination for every score (DeLong's test; Figure 3), with the largest gains for scores with lower baseline performance. MELD 3.0–sarcopenia showed the highest discrimination at 12 months (AUC 0.845). Pairwise comparisons between sarcopenia‐augmented scores were significant only between the MELD 3.0‐ and MELD‐Na‐sarcopenia versus ExPeCT‐ and CP‐sarcopenia (Figure S5). Harrell's C‐index values are reported in Table S3.

### 3.4 Risk Factors for Post‐TIPS OHE and Exploratory Profiles

**FIGURE 4:**
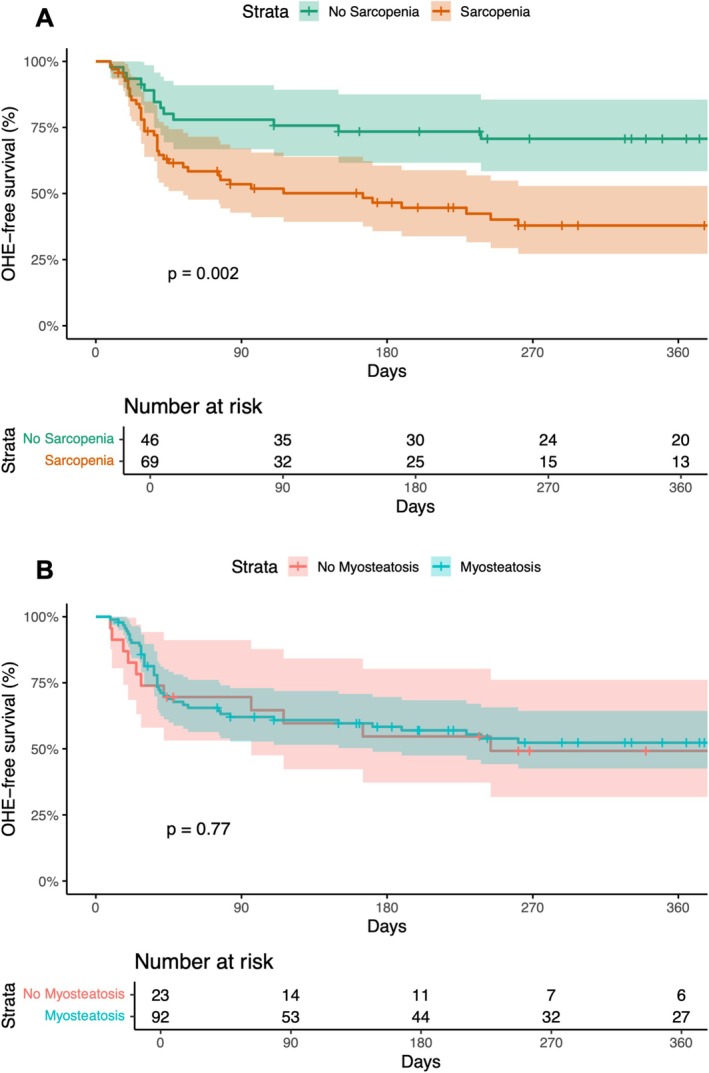
Kaplan–Meier estimates of time to first episode of OHE after TIPS, stratified by the presence of sarcopenia (A) and myosteatosis (B). Differences between groups were assessed using the log‐rank test. OHE, overt hepatic encephalopathy; TIPS, transjugular intrahepatic portosystemic shunt.

Fifty‐two patients (45%) developed OHE at a median 37 days (IQR 24–76) after TIPS (Figure S6). Cumulative OHE probability was higher in sarcopenic patients [62% (95% CI 47–73) vs. 29% (95% CI 14–42); log‐rank *p* = 0.002], whereas myosteatosis was not associated with OHE (log‐rank *p* = 0.770) (Figure 4). Accounting for death as a competing event, the 12‐month cumulative OHE incidence remained higher in sarcopenic patients [57% (44–67) vs. 28% (16–42); Grey's *p* = 0.004] (Figure S7). On multivariable Cox analysis, sarcopenia was independently associated with OHE (hazard ratio [HR] 2.06; 95% CI 1.07–3.96; *p* = 0.030), whereas underdilated TIPS reduced the risk (HR 0.55, 95% CI 0.31–0.96; *p* = 0.035) (Table 3). Findings were confirmed by competing‐risk Fine–Grey regression: sarcopenia (sHR 1.97; 1.03–3.76; *p* = 0.040) and underdilated TIPS (sHR 0.56; 0.32–0.98; *p* = 0.041) (Table S4).

**TABLE 3:** Univariable and multivariable Cox regression analyses of risk factors for post‐TIPS overt hepatic encephalopathy.

Variable	Univariable analysis		Multivariable analysis	
	HR (95% CI)	*p*	HR (95% CI)	*p*
**Male sex**	**2.15 (1.15–4.03)**	**0.017**	*1.85 (0.97–3.51)*	*0.061*
**Sarcopenia**	**2.62 (1.39–4.91)**	**0.003**	**2.06 (1.07–3.96)**	**0.030**
Myosteatosis	0.91 (0.47–1.76)	0.8	**—**	**—**
SATI	0.99 (0.99–1.00)	0.14	**—**	**—**
VATI	0.99 (0.98–1.01)	0.3	**—**	**—**
Diabetes	1.53 (0.88–2.64)	0.13	**—**	**—**
**TIPS underdilated** ^a^	**0.50 (0.29–0.87)**	**0.015**	**0.54 (0.31–0.96)**	**0.036**
Post‐TIPS PCPG < 10 mmHg	1.37 (0.79–2.39)	0.3	**—**	**—**
History of OHE	1.16 (0.56–2.38)	0.7	**—**	**—**
History of ascites^b^	1.50 (0.83–2.72)	0.2	0.98 (0.52–1.82)	0.938
*Total Bilirubin*	*1.28 (0.96–1.71)*	*0.090*	1.18 (0.87–1.61)	0.281
INR	1.00 (0.27–3.68)	> 0.9	—	—
Creatinine	1.01 (0.56–1.82)	> 0.9	—	—
*Serum sodium*	*0.95 (0.90–1.01)*	*0.091*	0.97 (0.92–1.03)	0.295
Albumin	1.22 (0.75–1.99)	0.4	—	—
CP score	1.07 (0.88–1.31)	0.5	—	—
MELD score	1.01 (0.94–1.08)	0.8	—	—
MELD‐Na score	1.03 (0.97–1.08)	0.3	—	—
MELD 3.0 score	1.02 (0.97–1.07)	0.49	—	—
FIPS score	1.02 (0.53–1.95)	> 0.9	—	—
ExPeCT score	1.12 (0.67–1.87)	0.7	—	—

*Note:* Time to first episode of overt hepatic encephalopathy was analysed using Cox proportional hazards regression. For each variable, hazard ratios (HR), 95% confidence intervals (CI), and *p* values are reported. Values in italics indicate *p* < 0.10, while values in bold indicate statistically significant predictors (*p* < 0.05). Variables with *p* < 0.10 at univariable analysis were entered into the multivariable Cox regression model. Clinically relevant covariates (sex and history of ascites) were included regardless of statistical significance.

Abbreviations: CP, Child Pugh; ExPeCT, Elderly Patients Calculator TIPS; FIPS, Freiburg index of post‐TIPS survival; INR, international normalised ratio; MELD, Model for End‐Stage Liver Disease; OHE, overt hepatic encephalopathy; PCPG, portocaval pressure gradient; SATI, subcutaneous adipose tissue index; TIPS, transjugular intrahepatic portosystemic shunt.

^a^ TIPS were defined “underdilated” when dilated with an angioplasty balloon‐catheter with a diameter less than or equal to 7 mm.

^b^ Ascites grade ≥ 2.

**FIGURE 5:**
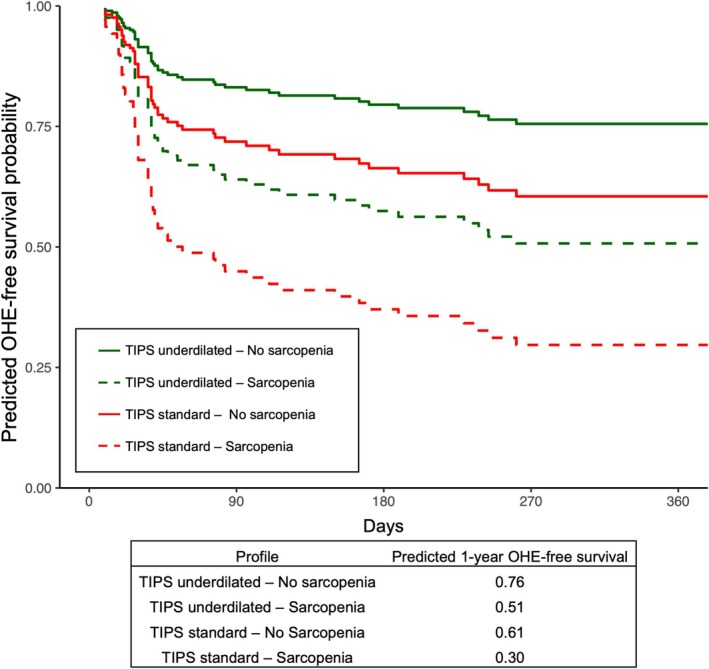
Predicted probabilities of 1‐year OHE‐free survival in older adult patients according to four risk profiles defined by sarcopenia status and TIPS dilation diameter. Solid green curve: Low‐risk profile (underdilated TIPS without sarcopenia); dashed green curve: Intermediate‐risk profile (underdilated TIPS with sarcopenia); solid red curve: Intermediate‐risk profile (standard‐diameter TIPS without sarcopenia); dashed red curve: High‐risk profile (standard‐diameter TIPS with sarcopenia). Dashed lines indicate the presence of sarcopenia, while solid lines indicate the absence of sarcopenia. OHE, overt hepatic encephalopathy; TIPS, transjugular intrahepatic portosystemic shunt.

In an exploratory analysis based on these dichotomous variables, four distinct 12‐month OHE risk profiles emerged (Figure 5). The most favourable profile—no sarcopenia combined with underdilated TIPS—was associated with a predicted OHE probability of 24%; the worst—sarcopenia with standard‐diameter TIPS—with 70%. Two intermediate profiles (no sarcopenia with standard TIPS and sarcopenia with underdilated TIPS) showed intermediate predicted risks (39% and 49%, respectively). These findings highlight the combined and opposing effects of sarcopenia and TIPS dilation diameter on post‐TIPS OHE risk and support their joint use for individualised risk stratification.

## 4 Discussion

The prevalence of cirrhosis among older adults has increased in recent years, driven by population aging, improved management of etiological factors and cirrhosis‐related complications and the rising incidence of MASLD‐related cirrhosis [31], decompensated cirrhosis is particularly prevalent in older patients [32]. Although TIPS is an established treatment for PH–related complications, evidence supporting its efficacy is largely derived from trials in younger populations (mean age ≈55 years) [33], leaving uncertainty regarding its safety and effectiveness in older adults, in whom LT is often unavailable. Physiological resilience to abrupt portal and systemic hemodynamic changes is reduced in older patients, in whom sarcopenia, frailty, and diminished cognitive and cardiac reserve may further increase the risk of post‐TIPS complications, including OHE and cardiac decompensation. The available literature, despite methodological limitations, often reports higher morbidity and mortality in older TIPS candidates [2, 34]. We previously reported [1] favourable survival outcomes in patients aged ≥ 70 years undergoing TIPS, suggesting that age alone should not be an a priori exclusion criterion; however, optimal selection criteria for this population remains a significant challenge.

Data on the predictive value of sarcopenia in cirrhosis are partially conflicting [11, 35]. In TIPS, some studies have highlighted sarcopenia as an independent risk factor for mortality [19, 36], although evidence—generally in non‐elderly patients—remains inconsistent [18, 37]. Moreover, sarcopenia is intrinsically linked to biological aging [14, 38], and its impact may be amplified by diminished cognitive reserve in older patients with cirrhosis.

In this multicenter cohort of 115 patients with cirrhosis aged ≥ 70 years who underwent TIPS for refractory ascites or secondary prophylaxis of variceal bleeding, sarcopenia was highly prevalent and independently associated with both post‐TIPS mortality and OHE, indicating that sarcopenia assessment may refine the identification of suitable TIPS candidates among older patients. Beyond its independent prognostic value, the integration of sarcopenia into established prognostic scores significantly enhances their discrimination for mortality. The MELD–sarcopenia score was first proposed in 2015 [7], and Bai et al. later validated MELD–sarcopenia specifically in the TIPS, identifying sarcopenia as an independent risk factor for post‐TIPS mortality in a predominantly non‐elderly population [36]. Similar results were subsequently confirmed, further supporting the prognostic relevance of combining liver‐severity scores with muscle mass assessment [21]. Our study expands this evidence to patients aged ≥ 70 years, showing that the prognostic value of sarcopenia is preserved in this more biologically vulnerable population and that its integration into commonly used prognostic scores provides meaningful incremental information for mortality risk stratification.

Sarcopenia‐adjusted models consistently showed improved discrimination compared with conventional scores. Predicted survival probabilities derived from MELD 3.0–sarcopenia profiles separated patients into distinct risk categories, both across increasing MELD 3.0 values and within each class according to sarcopenia status. Beyond mortality prediction, our data demonstrated a central role for sarcopenia in post‐TIPS OHE: multivariable analyses identified sarcopenia and TIPS dilation diameter as independent determinants of post‐TIPS OHE, with opposite effects (sarcopenia increased the risk; TIPS underdilation was protective). Because the cohort comprised older adults with substantial mortality, we confirmed these findings in a competing‐risk analysis. Although the sarcopenia–OHE association was slightly attenuated in the Fine–Grey model—as expected, given the higher competing risk of death in the sarcopenic group—the relationship remained statistically and clinically relevant. Overall, incorporating sarcopenia into prognostic assessment may improve stratification and support a more personalised approach to TIPS in older adults.

Myosteatosis, isolated or combined with sarcopenia, was also highly prevalent (80%), consistent with rates reported among older adults with other chronic conditions, including chronic kidney disease, heart failure, and cancer [15, 39, 40, 41, 42, 43]. Although myosteatosis and adipose‐tissue indices have been associated with worse outcomes in cirrhosis [44, 45], we found no significant association with mortality or OHE. A possible explanation is that these parameters—more rapidly modifiable after TIPS—may exert their prognostic impact through short‐term dynamic changes rather than baseline values. Mean MA and VATI in our cohort were markedly lower than in younger patients [30], likely reflecting both age and the cumulative impact of chronic liver disease. Prospective studies with serial body‐composition assessments are needed to clarify the prognostic role of myosteatosis and adipose‐tissue distribution in older patients with cirrhosis.

Several mechanisms may underlie the association between sarcopenia and adverse post‐TIPS outcomes. Beyond the inability to cope with abrupt hemodynamic shifts, sarcopenia directly contributes to OHE: skeletal muscle is a key alternative site for ammonia detoxification, and its depletion exacerbates hyperammonemia [16, 46]. Sarcopenia is also linked to systemic inflammation and immune dysfunction, predisposing to infections and clinical deterioration; these effects may be magnified in patients with reduced cardiac reserve and metabolic alterations, especially those with MASLD [47, 48].

The onset of OHE represents a highly burdensome complication for both patients and caregivers; in older adults, OHE can trigger a definitive loss of functional autonomy and substantially increase the risk of permanent institutionalisation.

Within this broader context of biological vulnerability, Kabelitz et al. [49] recently highlighted the prognostic relevance of frailty—measured by the Liver Frailty Index—in patients with cirrhosis undergoing elective TIPS. As the authors note, sarcopenia is not equivalent to frailty: frailty encompasses muscle quantity, dynamic muscle function, and cognitive decline. Sarcopenia and frailty represent distinct but complementary dimensions of the same underlying vulnerability: sarcopenia provides an objective morphological metric readily available from routine pre‐procedural CT, whereas frailty assessments capture functional and physiological decline. Integration of CT‐derived muscle parameters with comprehensive functional frailty assessments may represent the future benchmark for pre‐TIPS risk stratification in the geriatric population, supporting individualised decision‐making and procedural tailoring. Whether sarcopenia in older patients with cirrhosis can be effectively addressed through nutritional or exercise interventions as part of prehabilitation programs remains uncertain [46], and additional studies are required to clarify the reversibility of muscle depletion and identify potential therapeutic targets.

Strengths of this study include its multicenter design, the focus on older adults—an underrepresented population in the TIPS literature—and the objective evaluation of muscle mass and quality. Several limitations should be acknowledged. First, although data were prospectively collected, the retrospective design and stringent inclusion criteria may have introduced selection bias. Second, sarcopenic and non‐sarcopenic patients differed in BMI and sex distribution; despite adjustment for clinically relevant covariates in multivariable models, residual confounding cannot be excluded, and generalisability to populations with different ethnic backgrounds, BMI distributions, or aetiologies may be limited. Third, per RI‐TIPS protocol, no patients received primary OHE prophylaxis—a strategy that may differ from other centres and could affect the generalisability of our OHE‐incidence estimates. Fourth, baseline serum ammonia was not routinely collected in our registry, precluding calculation of recently developed predictive models such as the AMMON‐OHE score [50]. Fifth, post‐TIPS changes in sarcopenia and myosteatosis were unavailable, precluding assessment of dynamic changes over time. Finally, sarcopenia was integrated using established scores using a pragmatic, uniform approach rather than score‐specific recalibration; although intentional to preserve interpretability and the original structure of validated scores, larger studies are needed for formal recalibration or external validation.

In conclusion, in this multicenter cohort of patients with cirrhosis aged ≥ 70 years undergoing TIPS, sarcopenia was highly prevalent and independently associated with both mortality and OHE. Although established prognostic scores retained acceptable performance, incorporating sarcopenia consistently improved discrimination and refined outcome stratification. The combined assessment of sarcopenia and TIPS dilation diameter further identified clinically distinct OHE risk profile, highlighting the interplay between patient‐related vulnerability and procedural strategy. These findings support routine sarcopenia evaluation in older TIPS candidates and suggest that its integration into established prognostic tools may enhance individualised risk assessment and clinical decision‐making.

## Author Contributions

Angelica Ingravallo: data curation. Antonio Piscopo: data curation. Andrea Salomé Velasco Mayorga: data curation. Cristian Caporali: investigation. Davide Roccarina: writing – review and editing. Dario Saltini: methodology, formal analysis, investigation, data curation, writing – original draft, visualization. Francesco Ascari: investigation. Federico Banchelli: methodology, formal analysis. Federico Casari: investigation. Fabio Marra: supervision. Fabiola Milosa: data curation. Filippo Scianò: data curation. Filippo Schepis: conceptualization, methodology, writing – review and editing, project administration, supervision, funding acquisition. Francesco Vizzutti: writing – review and editing. Giovanni Battinelli: investigation, data curation. Gianmarco Falcone: investigation. Luigi Maruzzelli: investigation, data curation. Marcello Bianchini: investigation, data curation. Manuela Merli: supervision. Oliviero Riggio: supervision. Roberto Miraglia: investigation, data curation. Rosina Maria Critelli: data curation. Simone Di Cola: investigation, data curation. Silvia Nardelli: investigation, software, data curation. Stefania Gioia: data curation. Tomas Guasconi: data curation.

## Conflicts of Interest

Dario Saltini is supported by grants (CUP E53D23013280006) from the PRIN project (Prot. 2022N2KJAM) by the Italian Ministry of University and Research (MUR); Filippo Scianò is supported by grants from the Italian Ministry of Health under the “Ricerca Finalizzata” program (RF‐2021‐12372399); Filippo Schepis has served as a consultant for Cook Medical and Echosens; Dario Saltini, Filippo Schepis and Francesco Vizzutti received honoraria from Cook Medical and W. L. Gore & Associates. Angelica Ingravallo, Antonio Piscopo, Andrea Salomé Velasco Mayorga, Cristian Caporali, Davide Roccarina, Francesco Ascari, Federico Banchelli, Federico Casari, Fabio Marra, Fabiola Milosa, Giovanni Battinelli, Gianmarco Falcone, Luigi Maruzzelli, Marcello Bianchini, Manuela Merli, Oliviero Riggio, Roberto Miraglia, Rosina Maria Critelli, Simone Di Cola, Stefania Gioia, Silvia Nardelli, Tomas Guasconi: no conflicts to declare.

## Supporting information


**Table S1:** Univariable Cox regression analysis for post‐TIPS mortality.
**Table S2:** Predicted post‐TIPS survival probabilities in older adult patients according to three MELD 3.0–based risk profiles at 12‐ and 24‐months.
**Table S3:** Harrell's C‐index of sarcopenia‐augmented prognostic scores.
**Table S4:** Univariable and multivariable Fine–Grey analysis predicting the overall cumulative incidence of post‐TIPS OHE, considering death as a competing event.
**Figure S1:** Study flowchart illustrating patient identification, screening and inclusion. A total of 516 patients with cirrhosis undergoing TIPS at one of the participating centers (Florence, Modena, Palermo, and Rome) between June 2015 and March 2023 were included. Of these, 182 were aged ≥ 70 years, and 134 had refractory ascites or secondary prophylaxis of variceal bleeding as the indication for TIPS. For 19 patients, no data on muscle condition were available. In total, 115 patients met all the inclusion criteria.
**Figure S2:** Distribution of sarcopenia and myosteatosis among the study cohort.
**Figure S3:** Scatter plots illustrating the correlations between muscle and adipose tissue indices. Specifically, correlation between: (A) muscle attenuation (MA) and subcutaneous adipose tissue index (SATI); (B) muscle attenuation (MA) and visceral adipose tissue index (VATI); (C) skeletal muscle index (SMI) and subcutaneous adipose tissue index (SATI); (D) skeletal muscle index (SMI) and visceral adipose tissue index (VATI).
**Figure S4:** Kaplan–Meier estimates of post‐TIPS overall survival for the entire study population.
**Figure S5:** Pairwise comparisons of AUROC values among sarcopenia‐augmented prognostic scores. Differences between AUROCs were assessed using DeLong's test. Pairwise comparisons that did not reach statistical significance are not shown.
**Figure S6:** Kaplan–Meier estimates of time to first episode of OHE after TIPS.
**Figure S7:** Cumulative incidence functions for OHE after TIPS, with death as competing event. Curves are stratified by presence of (A) sarcopenia and (B) myosteatosis. *p*‐value from Grey's test.

## Data Availability

All data supporting the findings of this study are available within the article and its Supporting Information. The individual‐level dataset is not publicly available due to privacy restrictions, but can be made available by the corresponding author upon reasonable request for academic and research purposes.
