# Supplementary material for: Sarcopenia Predicts Mortality and Hepatic Encephalopathy After TIPS in Older Adults With Cirrhosis and Improves Prognostic Scores
Source: Liver Int. 2026 Jun 7;46(7):e70733. doi: 10.1111/liv.70733 (PMC13243813; doi:10.1111/liv.70733)
Supplement: Supplementary file 1 — Table S1: Univariable Cox regression analysis for post‐TIPS mortality. Table S2: Predicted post‐TIPS survival probabilities in older adult patients according to three MELD 3.0–based risk profiles at 12‐ and 24‐months. Table S3: Harrell's C‐index of sarcopenia‐augmented prognostic scores. Table S4: Univariable and multivariable Fine–Grey analysis predicting the overall cumulative incidence of post‐TIPS OHE, considering death as a competing event. Figure S1: Study flowchart illustrating patient identification, screening and inclusion. A total of 516 patients with cirrhosis undergoing TIPS at one of the participating centers (Florence, Modena, Palermo, and Rome) between June 2015 and March 2023 were included. Of these, 182 were aged ≥ 70 years, and 134 had refractory ascites or secondary prophylaxis of variceal bleeding as the indication for TIPS. For 19 patients, no data on muscle condition were available. In total, 115 patients met all the inclusion criteria. Figure S2: Distribution of sarcopenia and myosteatosis among the study cohort. Figure S3: Scatter plots illustrating the correlations between muscle and adipose tissue indices. Specifically, correlation between: (A) muscle attenuation (MA) and subcutaneous adipose tissue index (SATI); (B) muscle attenuation (MA) and visceral adipose tissue index (VATI); (C) skeletal muscle index (SMI) and subcutaneous adipose tissue index (SATI); (D) skeletal muscle index (SMI) and visceral adipose tissue index (VATI). Figure S4: Kaplan–Meier estimates of post‐TIPS overall survival for the entire study population. Figure S5: Pairwise comparisons of AUROC values among sarcopenia‐augmented prognostic scores. Differences between AUROCs were assessed using DeLong's test. Pairwise comparisons that did not reach statistical significance are not shown. Figure S6: Kaplan–Meier estimates of time to first episode of OHE after TIPS. Figure S7: Cumulative incidence functions for OHE after TIPS, with death as competing event. Curves are stra [file LIV-46-0-s001.docx]

**Supplementary Material**

**Title:** Sarcopenia predicts mortality and hepatic encephalopathy after TIPS in older adults with cirrhosis and improves prognostic scores

**Authors**: Dario Saltini*^1^, Silvia Nardelli*^2^, Roberto Miraglia^3,4^, Davide Roccarina^5^, Stefania Gioia^2^, Federico Banchelli^6^, Luigi Maruzzelli^3,4^, Cristian Caporali^7^, Gianmarco Falcone^8^, Marcello Bianchini^1^, Tomas Guasconi^1^, Angelica Ingravallo^5^, Simone Di Cola^2^, Rosina Maria Critelli^9^, Fabiola Milosa^9^, Antonio Piscopo^1,10^, Federico Casari^7^, Andrea Salome Velasco Mayorga^1,10^, Filippo Scianò^1,10^, Giovanni Battinelli^7^, Francesco Ascari^7^, Oliviero Riggio^2^, Fabio Marra^5^, Manuela Merli^2^, Francesco Vizzutti^5,11^, Filippo Schepis^1,10^

*: These Authors shares first authorship

**Affiliations:**

1. Severe Liver Diseases (M.E.C.) Departmental Unit, Department of Medical Specialties, University Hospital of Modena "Policlinico", University of Modena and Reggio Emilia, European Reference Network on Rare Liver Disorders (ERN Rare-Liver), Modena, Italy.
2. Department of Translational and Precision Medicine, Sapienza University of Rome, Rome, Italy.
3. IRCCS ISMETT, Radiology Service, Palermo, Italy.
4. UPMC Italy, Palermo, Italy
5. Department of Experimental and Clinical Medicine, University of Florence, Florence, Italy.
6. Medical Statistics Unit, Department of Medical and Surgical Sciences, University of Modena and Reggio Emilia, Modena, Italy.
7. Division of Interventional Radiology, University Hospital of Modena "Policlinico", Modena, Italy.
8. Department of Radiology, Interventional Radiology Unit, Careggi Hospital, Florence, Italy.
9. EpatoGastro Lab – RBA-Labs CHIMOMO Department – University of Modena and Reggio Emilia, Modena, Italy.
10. Gastroenterology Unit, University Hospital of Modena "Policlinico", University of Modena and Reggio Emilia, Modena, Italy.
11. Portal Hypertension Departmental Unit, Department of Experimental and Clinical Medicine, University of Florence, Florence, Italy.

**Table of Contents:**

Supplementary Methods

Supplementary Tables (1-4)

Supplementary Figures (1-7)

**Supplementary Methods**

**Calculation of prognostic scores**

All prognostic scores were calculated using the original formulas as reported in the respective reference publications. Laboratory values obtained within 5 days prior to TIPS placement were used for score calculation.

Model for End-Stage Liver Disease (MELD) was calculated according to the original formula:

$$\text{MELD}=3.78\times\ln(\text{bilirubin [mg/dL]})+11.2\times\ln(\text{INR})+9.57\times\ln(\text{creatinine [mg/dL]})+6.43$$

MELD-Na was calculated using the following equation, with serum sodium values capped between 125 and 137 mmol/L:

$$\text{MELD-Na}=\text{MELD}+1.32\times(137-\text{Na})-[0.033\times\text{MELD}\times(137-\text{Na})]$$

MELD 3.0 was calculated according to the updated formula incorporating sex, serum albumin, bilirubin, creatinine, INR, and serum sodium, as previously published. Sex was coded as a binary variable, with female sex assigned a coefficient as specified in the original model:

MELD 3.0 = 1.33 (if female) + [4.56 x log_e_(bilirubin)] + [0.82 x (137 – Na)] – [0.24 x (137 – Na) x log_e_(bilirubin)] + [9.09 x log_e_(INR)] + [11.14 x log_e_(creatinine)] + [1.85 x (3.5 – albumin)] – [1.83 x (3.5 – albumin) x log_e_(creatinine)] + 6

Freiburg Index of Post-TIPS Survival (FIPS) was calculated using the published formula incorporating age, serum bilirubin, serum creatinine, and INR:

Linear Predictor = 1.43 x log10(bilirubin) - 1.71 x 1/creatinine + 0.02 x age - 0.02 x albumin +0.81

Child–Pugh (CP) score was calculated by summing points assigned to serum bilirubin, serum albumin, INR, presence of ascites, and hepatic encephalopathy, according to standard definitions, yielding a total score ranging from 5 to 15.

Elderly Patients Calculator TIPS (ExPeCT) score was calculated using the dedicated webapp (https://promisepa.shiny apps.io/TIPS/) modality “Older Adults (age equal or higher than 70 years)”.

**Supplementary Tables**

**Table S1. Univariable Cox regression analysis for post-TIPS mortality.**

|  | **Univariable analysis** | |
| --- | --- | --- |
| **Variable** | **HR (95% CI)** | **p-value** |
| Male sex | 1.32 (0.75–2.32) | 0.3 |
| **Sarcopenia** | **2.34 (1.27–4.32)** | **0.006** |
| Myosteatosis | 1.17 (0.60–2.29) | 0.6 |
| SATI, cm^2^/m^2^, (IQR) | 1.00 (1.00–1.01) | 0.5 |
| VATI cm^2^/m^2^, (IQR) | 1.00 (0.98–1.01) | 0.6 |
| MASLD* | 1.35 (0.77–2.38) | 0.3 |
| TIPS indication** | 0.67 (0.39–1.16) | 0.15 |
| TIPS underdilated*** | 0.80 (0.47–1.35) | 0.4 |
| **History of clinical ascites** | **2.10 (1.14–3.87)** | **0.017** |
| **Total Bilirubin, mg/dl** | **1.61 (1.26–2.06)** | **<0.001** |
| **INR** | **1.24 (1.10–1.40)** | **<0.001** |
| **Creatinine, mg/dl** | **2.15 (1.27–3.64)** | **0.005** |
| **Serum sodium, mmol/mol** | **0.93 (0.88–0.99)** | **0.021** |
| Albumin, g/dl | 0.89 (0.57–1.40) | 0.6 |
| **CP score** | **1.40 (1.16–1.68)** | **<0.001** |
| **MELD score** | **1.15 (1.08–1.22)** | **<0.001** |
| **MELD-Na score** | **1.12 (1.06–1.17)** | **<0.001** |
| **MELD 3.0 score** | **1.11 (1.06-1.16)** | **<0.001** |
| **FIPS score** | **2.89 (1.70–4.90)** | **<0.001** |
| **ExPeCT score** | **2.17 (1.43–3.29)** | **<0.001** |

* Reference: alcohol liver-related etiology. ** Reference: indication for secondary prophylaxis of variceal bleeding.*** TIPS were defined “underdilated” when dilated with an angioplasty balloon-catheter with a diameter less than or equal to 7 mm.

CP; Child Pugh; ExPeCT, Elderly Patients Calculator TIPS; FIPS, Freiburg index of post-TIPS survival; HCC, hepatocellular carcinoma; MALSD, Metabolic dysfunction-associated steatotic liver disease; MELD, Model for End-Stage Liver Disease; SATI, subcutaneous adipose tissue index; TIPS, transjugular intrahepatic portosystemic shunt; VATI, visceral adipose tissue index.

**Table S2. Predicted post-TIPS survival probabilities in older adult patients according to three MELD 3.0–based risk profiles at 12- and 24-months.**

| **Profile** | **Risk group** | **Sarcopenia** | **Time (months)** | **Predicted survival** |
| --- | --- | --- | --- | --- |
| Low risk – no sarcopenia | MELD 3.0 = 10 | No | 12 | 0.873 |
| Low risk – sarcopenia | MELD 3.0 = 10 | Yes | 12 | 0.744 |
| Intermediate risk – no sarcopenia | MELD 3.0 = 13 | No | 12 | 0.835 |
| Intermediate risk – sarcopenia | MELD 3.0 = 13 | Yes | 12 | 0.677 |
| High risk – no sarcopenia | MELD 3.0 = 18 | No | 12 | 0.752 |
| High risk – sarcopenia | MELD 3.0 = 18 | Yes | 12 | 0.539 |
| Low risk – no sarcopenia | MELD 3.0 = 10 | No | 24 | 0.829 |
| Low risk – sarcopenia | MELD 3.0 = 10 | Yes | 24 | 0.667 |
| Intermediate risk – no sarcopenia | MELD 3.0 = 13 | No | 24 | 0.781 |
| Intermediate risk – sarcopenia | MELD 3.0 = 13 | Yes | 24 | 0.586 |
| High risk – no sarcopenia | MELD 3.0 = 18 | No | 24 | 0.676 |
| High risk – sarcopenia | MELD 3.0 = 18 | Yes | 24 | 0.428 |

MELD, Model for End-Stage Liver Disease.

**Table S3. Harrell’s C-index of sarcopenia-augmented prognostic scores.**

| **Model** | **C-index** | **SE** | **N° events (death)** | **Total cohort** |
| --- | --- | --- | --- | --- |
| MELD 3.0-Sarcopenia | 0.753 | 0.06 | 56 | 115 |
| MELD-Na-Sarcopenia | 0.752 | 0.06 | 56 | 115 |
| MELD-Sarcopenia | 0.742 | 0.06 | 56 | 115 |
| FIPS-Sarcopenia | 0.730 | 0.07 | 56 | 115 |
| CP-Sarcopenia | 0.717 | 0.08 | 56 | 115 |
| ExPeCT-Sarcopenia | 0.707 | 0.08 | 56 | 115 |

CP; Child Pugh; ExPeCT, Elderly Patients Calculator TIPS; FIPS, Freiburg index of post-TIPS survival; MELD, Model for End-Stage Liver Disease;

**Table S4.** Univariable and multivariable Fine–Gray analysis predicting the overall cumulative incidence of post-TIPS OHE, considering death as a competing event.

|  | **Univariable analysis** | | **Multivariable analysis** | | | |
| --- | --- | --- | --- | --- | --- | --- |
|  |  | | **Model 1** | | **Model 2** | |
| **Variable HR** | **sHR (95% CI)** | **p-value** | **sHR (95% CI)** | **p-value** | **sHR (95% CI)** | **p-value** |
| **Male sex** | **2.15 (1.14–4.06)** | **0.019** | *1.93 (0.98–3.80)* | *0.057* | *1.94 (0.99–3.81)* | *0.053* |
| **Sarcopenia** | **2.43 (1.30–4.55)** | **0.006** | **1.97 (1.03–3.76)** | **0.040** | **1.97 (1.03–3.76)** | **0.040** |
| Myosteatosis | 0.88 (0.45–1.72) | 0.72 | **-** | **-** | **-** | **-** |
| SATI | 0.99 (0.99-1.00) | 0.15 | **-** | **-** | **-** | **-** |
| VATI | 0.99 (0.98-1.00) | 0.23 | **-** | **-** | **-** | **-** |
| Diabetes | 1.46 (0.85–2.52) | 0.17 | **-** | **-** | **-** | **-** |
| **TIPS underdilated*** | **0.53 (0.31–0.92)** | **0.023** | **0.56 (0.32–0.98)** | **0.041** | **0.59 (0.31–0.99)** | **0.044** |
| Post-TIPS PCPG <10 mmHg | 1.45 (0.83–2.53) | 0.19 | **-** | **-** | **-** | **-** |
| History of OHE | 1.17 (0.56–2.48) | 0.67 | **-** | **-** | **-** | **-** |
| History of ascites** | 1.38 (0.77–2.46) | 0.28 | **-** | **-** | 0.96 (0.53-1.73) | 0.89 |
| Total Bilirubin | 1.27 (0.91–1.77) | 0.16 | **-** | **-** | 1.20 (0.80-1.80) | 0.38 |
| INR | 0.85 (0.23–3.11) | 0.81 | **-** | **-** | **-** | **-** |
| Creatinine | 0.89 (0.51–1.54) | 0.67 | **-** | **-** | **-** | **-** |
| Serum sodium | 0.96 (0.91–1.01) | 0.11 | **-** | **-** | 0.97 (0.92-1.02) | 0.27 |
| Albumin | 1.18 (0.71–1.96) | 0.52 | **-** | **-** | **-** | **-** |
| CP score | 1.05 (0.85–1.30) | 0.66 | **-** | **-** | **-** | **-** |
| MELD score | 1.01 (0.94–1.08) | 0.73 | **-** | **-** | **-** | **-** |
| MELD-Na score | 1.03 (0.98–1.08) | 0.32 | **-** | **-** | **-** | **-** |
| MELD 3.0 score | 1.00 (0.95-1.05) | >0.99 | **-** | **-** | **-** | **-** |
| FIPS score | 0.90 (0.46–1.73) | 0.75 | **-** | **-** | **-** | **-** |
| ExPeCT score | 0.80 (0.49–1.32) | 0.39 | **-** | **-** | **-** | **-** |

TIPS were defined “underdilated” when dilated with an angioplasty balloon-catheter with a diameter less than or equal to 7 mm. **Ascites grade $\geq2$.

Time to first episode of OHE was analyzed in a competing risk framework, with death as a competing event. For each variable, subdistribuzion hazard ratios (sHR), 95% confidence intervals (CI), and p values are reported. Variables with p<0.10 at univariable analysis were entered into the multivariable model 1, together with clinically relevant covariates (history of ascites, total bilirubin and serum sodium) in model 2.

CP, Child Pugh; ExPeCT, Elderly Patients Calculator TIPS; FIPS, Freiburg index of post-TIPS survival; INR, International Normalized Ratio; MELD, Model for End-Stage Liver Disease; OHE, Overt Hepatic Encephalopathy; PCPG, Portocaval Pressure Gradient; SATI, Subcutaneous Adipose Tissue Index; TIPS, Transjugular Intrahepatic Portosystemic Shunt.

**Supplementary Figures**

**Fig. S1. Study flowchart illustrating patient identification, screening and inclusion.**

A total of 516 patients with cirrhosis undergoing TIPS at one of the participating centers (Florence, Modena, Palermo, and Rome) between June 2015 and March 2023 were included. Of these, 182 were aged $\geq$70 years, and 134 had refractory ascites or secondary prophylaxis of variceal bleeding as the indication for TIPS. For 19 patients, no data on muscle condition were available. In total, 115 patients met all the inclusion criteria.

TIPS, Transjugular Intrahepatic Portosystemic Shunt; CT, Computed Tomography; RI-TIPS, TIPS-Italian Registry.

**Fig. S2. Distribution of sarcopenia and myosteatosis among the study cohort.**


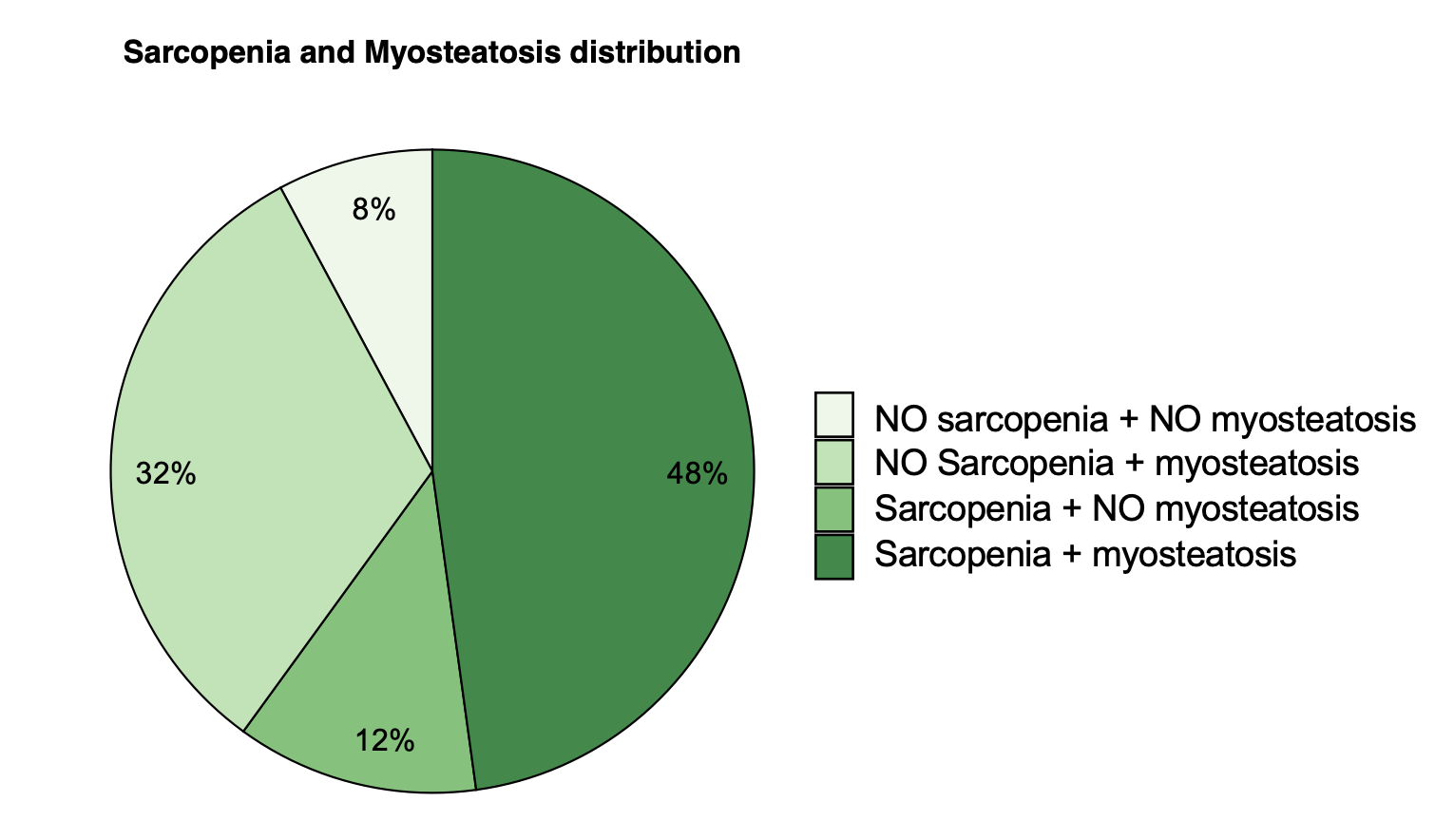


**Fig. S3. Scatter plots illustrating the correlations between muscle and adipose tissue indices**.

Specifically, correlation between: A) muscle attenuation (MA) and subcutaneous adipose tissue index (SATI); B) muscle attenuation (MA) and visceral adipose tissue index (VATI); C) skeletal muscle index (SMI) and subcutaneous adipose tissue index (SATI); D) skeletal muscle index (SMI) and visceral adipose tissue index (VATI).

Correlations were assessed using Spearman’s rank correlation coefficient (r). Locally estimated scatterplot smoothing (LOESS) curves illustrate the overall trend of the associations.

**Fig. S4. Kaplan–Meier estimates of post-TIPS overall survival for the entire study population.**

TIPS, transjugular intrahepatic portosystemic shunt.

**Fig. S5. Pairwise comparisons of AUROC values among sarcopenia-augmented prognostic scores.**

Differences between AUROCs were assessed using DeLong’s test. Pairwise comparisons that did not reach statistical significance are not shown**.**

AUROC, area under the receiver operating characteristic curve; CP, Child–Pugh score; ExPeCT, Elderly Patients Calculator TIPS; FIPS, Freiburg Index of Post-TIPS Survival; MELD, Model for End-Stage Liver Disease; TIPS, transjugular intrahepatic portosystemic shunt.

**Fig. S6. Kaplan–Meier estimates of time to first episode of OHE after TIPS.**

OHE, overt hepatic encephalopathy; TIPS, transjugular intrahepatic portosystemic shunt.

**Figure S7. Cumulative incidence functions for OHE after TIPS, with death as competing event. Curves are stratified by presence of (A) sarcopenia and (B) myosteatosis. P-value from Gray's test.**

**
A)**

**B)**

OHE, overt hepatic encephalopathy; TIPS, transjugular intrahepatic portosystemic shunt.
